# Mechanobiological responses of astrocytes in optic nerve head due to biaxial stretch

**DOI:** 10.1186/s12886-022-02592-8

**Published:** 2022-09-16

**Authors:** Zhiwen Li, Fan Peng, Zhicheng Liu, Shanshan Li, Lin Li, Xiuqing Qian

**Affiliations:** 1 School of Biomedical Engineering, Capital Medical University, No. 10 Xitoutiao, Youanmen Street, Fengtai District, Beijing, 100069 China; 2 Beijing Key Laboratory of Fundamental Research On Biomechanics in Clinical Application, Beijing, 100069 China

**Keywords:** Astrocytes, Biaxial stretch, Mechanobiological responses, Proteomics, Optic nerve head

## Abstract

**Background:**

Elevated intraocular pressure (IOP) is the main risk factor for glaucoma, which might cause the activation of astrocytes in optic nerve head. To determine the effect of mechanical stretch on the astrocytes, we investigated the changes in cell phenotype, proteins of interest and signaling pathways under biaxial stretch.

**Method:**

The cultured astrocytes in rat optic nerve head were stretched biaxially by 10 and 17% for 24 h, respectively. Then, we detected the morphology, proliferation and apoptosis of the stretched cells, and performed proteomics analysis. Protein expression was analyzed by Isobaric tags for relative and absolute quantification (iTRAQ) mass spectrometry. Proteins of interest and signaling pathways were screened using Gene Ontology enrichment analysis and pathway enrichment analysis, and the results were verified by western blot and the gene-chip data from Gene Expression Omnibus (GEO) database.

**Result:**

The results showed that rearrangement of the actin cytoskeleton in response to stimulation by mechanical stress and proliferation rate of astrocytes decreased under 10 and 17% stretch condition, while there was no significant difference on the apoptosis rate of astrocytes in both groups. In the iTRAQ quantitative experiment, there were 141 differential proteins in the 10% stretch group and 140 differential proteins in the 17% stretch group. These proteins include low-density lipoprotein receptor-related protein (LRP6), caspase recruitment domain family, member 10 (CARD10), thrombospondin 1 (THBS1) and tetraspanin (CD81). The western blot results of LRP6, THBS1 and CD81 were consistent with that of iTRAQ experiment. ANTXR2 and CARD10 were both differentially expressed in the mass spectrometry results and GEO database. We also screened out the signaling pathways associated with astrocyte activation, including Wnt/β–catenin pathway, NF-κB signaling pathway, PI3K-Akt signaling pathway, MAPK signaling pathway, Jak-STAT signaling pathway, ECM-receptor interaction, and transforming growth factor-β (TGF-β) signaling pathway.

**Conclusion:**

Mechanical stimulation can induce changes in cell phenotype, some proteins and signaling pathways, which might be associated with astrocyte activation. These proteins and signaling pathways may help us have a better understanding on the activation of astrocytes and the role astrocyte activation played in glaucomatous optic neuropathy.

**Supplementary Information:**

The online version contains supplementary material available at 10.1186/s12886-022-02592-8.

## Introduction

Glaucoma is a neurodegenerative ocular disease characterized by regional visual loss owing to irreversible apoptotic death of retinal ganglion cells at the optic nerve head (ONH) [1]. Elevated intraocular pressure (IOP) is the main risk factor for glaucoma [2]. Astrocytes are major glial cells that provide structural and functional support in optic nerve head (ONH). In response to elevated IOP, they remodel and become reactive, inducing the changes in morphology, gene expression, and function [3–5]. It is found that the reactivity of ONH astrocyte emerged before the damage of optic nerve [6]. Therefore, it is important to research the mechanobiological responses of ONH astrocyte cells.

ONH astrocyte reactivity to elevated IOP includes alterations of morphology and protein expression. The cell body of astrocytes in the prelaminar region became hypertrophy [3], and processes became longer [4] and thicker [5]. In the lamina cribrosa, the astrocytes showed round-shaped cell bodies and their processes disappeared [7]. At the same time, astrocytes in the myelination transition zone extend new processes along the longitudinal axis of the optic nerve and invade axonal bundles in the nerve head [8]. Lozano [9] found that the nucleus number in lamina cribrosa increased significantly under chronic intraocular hypertension, and astrocytes accounted for the majority of the proliferating cells. In addition to morphological changes, glial fibrillary acidic protein (GFAP) expression in ONH astrocytes and prelaminar region increased with chronic intraocular hypertension and axonal injury [3, 5]. Actin and GFAP in optic nerve head were arranged horizontally under the elevated IOP, suggesting that the processes of astrocytes were also rearranged with the increase of IOP [5, 10].

Previous studies have shown that the activation of astrocytes was related to some signaling pathways. STAT3 signaling is an important mediator of various aspects of the reactive phenotype within optic nerve astrocytes [3, 11]. STAT3 knockout mice showed attenuated astrocytes were hypertrophy and reactive remodeling, and astrocytes basically maintained a cellular structure of tissue and glial ducts. Tezel et al. [12] verified that astrocytes would experience cell activation and immune/inflammatory reactions under high intraocular pressure using mass spectrometry. These responses included up-regulation of a number of immune mediators/regulators linked to TNF-a/TNFR signaling, nuclear factor kappa-B (NF-κB) activation, autophagy regulation, and inflammasome assembly.

Mechanical factors may lead to the changes of astrocytes protein expression. Albalawi et al. [13] used FX-5000 T system (Flexcell International Corp.) to stretch astrocytes with 16% for 4 h at 0.3 Hz, confirming that astrocytes were involved in the upregulation of retinal cytokine IL-6 and the release of P2X7 receptor. Beckel [14] designed an apparatus to stretch astrocytes under an equibiaxial strain of 5% at a 0.3 Hz cycle. They found that ATP release from astrocytes was in response to mechanical strain and that Pannexin 1 was the major efflux pathway. Mulvihill et al. [15] developed a three-dimension collagen gel culture system to mimic features of ONH deformation due to elevated IOP. They found that compressive loading of astrocyte-seeded collagen gels led to cell alignment perpendicular to the direction of strain, and increased astrocyte activation, such as the changes of GFAP, vimentin, and S100B levels.

Therefore, different mechanical stimulates may cause changes in protein expression of astrocytes and activation of some signaling pathways, which may be closely related to the activation of astrocytes in glaucoma patients. However, the mechanism affecting astrocyte activation is very complex. In this study, we researched the mechanobiological responses of astrocytes in optic nerve head under biaxial stretch, including cell phenotype and protein expression.

## Materials and methods

### Cell lines

The primary culture rats ONH astrocytes were purchased from Procell Life Science & Technology Co (Wuhan, China). Then the cells were cultured at 37 °C and 5% CO2 in Dulbecco Modified Eagle Medium F/12 (GIBCO, United States) supplemented with 10% fetal bovine serum (FBS, GIBCO, United States) and 1% penicillin/ streptomycin (Hyclone, United States). The cells were grown to confluence and split in this media until the fourth generation to obtain enough viable cells for the experiment. A third cell line was grown to be used for validation purposes using immunohistochemistry.

### Immunohistochemistry

Cells from the third cell line were washed twice with phosphate buffered saline (PBS), fixed with -20 °C pre-cooled methanol for 5 min, and then blocked with 5% BSA, 0.3 M glycine (PBST) for 2 h at room temperature (RT). Then, the cells were incubated with Rabbit polyclonal to GFAP (1:1000; Abcam, United Kingdom), Vimentin (1:100; CST, United States), Connexin43(1:250; Abcam, United Kingdom), Aquaporin 4 (1:300; Abcam, United Kingdom), OSP (1:250; Bioss, United Kingdom) and F4/80 (1:50; Abcam, United Kingdom) in dark overnight at 4 °C. Following washes with PBS 3 times, the cells were incubated with Goat polyclonal Secondary Antibody to Rabbit IgG—H&L (1:500; Abcam, United States) for 2 h at 37 °C in dark. Subsequently, cells were incubated with DAPI for 5 min at RT in dark.

### Mechanical stretch

The cells at the fourth passage were seeded onto 6-well Flexcell plates (biaxial six-hole plate # bf-3001c, Flexcell International Corp., United States) and allowed to grow to confluence for 3–4 days. They were divided into three groups: the 10% stretch group, the 17% stretch group and the control group. All cells were serum deprived for 24 h prior to stretch. After washed for 3 times, two stretch group cells were stretched using Flexcell FX-5000 T tension system (Flexcell International Corp., United States) under biaxial cyclic loading with 1 Hz cycle of 0 to 10%, or 0 to 17% for 24 h, respectively. The control cells were cultured on similar plates and kept in the same incubator without stretch. Three biological replications for the cells under different stretch conditions were conducted for subsequent experiments.

### Cytoskeleton staining

Different groups of cells were fixed with 4% paraformaldehyde for 10 min at RT, and then permeabilized with 0.5% Triton X-100 (Beyotime, China) for 10 min. Then, the cells were incubated with Alexa Fluor 568 Phalloidin (1:200, CST, United States) for 30 min. After incubation with DAPI (4’, 6-diamidino-2-phenylindole, Abcam, United States) for 5 min, the cells were mounted by coverslips and observed by a Fluorescence microscope (Axio Observer D1, Zeiss, Germany).

### Cell apoptosis

After digestion with EDTA-free trypsin, the cells were re-suspended and then centrifuged at 1000 rpm for 5 min. Next, the supernatant was discarded. After added 1 mL PBS, and the cells were centrifuged. Then supernatant was discarded. Following washed with PBS twice, the cells were suspended again with 1:10 diluted binding buffer solution. Adjust the concentration of cells to 1 × 10^6^/mL. Using Annexin V-FITC/PI Apoptosis Detection Kit (P04D01, Beyotime, China), we added Annexin V-FITC 5 μL and Propyl iodide 10 μL to the 100μL cell suspension. After the mixture was incubated at RT and away from light for 15 min, PBS was added to the reaction tube and cell apoptosis was analyzed by flow cytometry (ACEA NovoCyte 3130, ACEA Biosciences Inc., United States) within 1 h.

### Cell proliferation

Cell proliferation was detected using EdU Proliferation Detection Kit (C10310, Ribobio, Guangzhou, China). After incubated in EdU (5-ethynyl-2’-deoxyuridine) medium for 24 h, the cells were fixed with 4% paraformaldehyde at RT for 10 min. Then, cells were incubated in 2 mg/ml Glycine for 5 min. Following washed with PBS 3 times, the cells were incubated successively with osmotic agent (0.5% PBS of TritonX-100), 1X Apollo® staining reaction solution and osmotic agent (0.5% PBS of TritonX-100). Subsequently, cells were incubated with DAPI for 5 min at RT in dark, followed by 3-time washes in PBS. Three wells were selected for each group of conditions, and three areas per well were selected for fluorescence imaging (*n* = 9). Afterwards, the number of proliferated cells and the number of cells nuclear stained by DAPI were counted by ImageJ.

### LC-MS/MS

Stretched rat astrocytes were collected and lysed for protein extraction. SDS-PAGE was used to confirm protein quality. Protein concentration was determined by Bradford test, and then all the samples were labeled with iTRAQ reagents. For iTRAQ labeling, proteins collected from each sample were reduced with 10 mM DTT at 56 °C for 1 h, and alkylated with 55 mM iodoacetamide in darkness at RT for 45 min. Protein solution was precipitated with cold acetone at a ratio of 1:5 at − 20 °C for 30 min and centrifuged at 25,000 g at 4 °C for 15 min. The protein precipitate obtained after centrifugation was air-dried and dissolved in lysis buffer without SDSL3. After determining protein concentration, 100 μg protein solutions were subjected to tryptic hydrolysis at a ratio of enzyme to protein of 1:20 at 37 °C for 2 h. After desalting and freeze drying, the peptides were resolved with 0.5 M TEAB added to the corresponding iTRAQ labeling reagent. Shimadzu LC-20AB liquid phase system was used to separate the sample in liquid phase with a separation column, Gemini 5 μm C18, 4.6 × 250 mm. The elution peak was monitored at a wavelength of 214 nm and the samples were combined according to the chromatographic elution peak map to obtain 20 components, which were then freeze-dried. The dried peptide samples were reconstituted, centrifuged at 20,000 g for 10 min. And the supernatant was taken for injection. Separation was performed by Thermo UltiMate 3000 UHPLC. The sample was first enriched in trap column and desalted, and then entered a self-packed C18 column and separated at a flow rate of 300 nL/min. The nano liter liquid phase separation end was directly connected to the mass spectrometer. The peptides separated by liquid phase chromatography were ionized by a nanoESI source and then passed to a tandem mass spectrometer Q-Exactive HF X (Thermo Fisher Scientific, United States) for DDA (data-dependent acquisition) mode detection.

### Data processing and analysis

Relative quantification and protein identification were performed with IQuant software independently developed by BGI [16] (The Beijing Genomics Institute, China). This software integrates Mascot Percolator, a well performing machine learning method for rescoring database search results, to provide reliable significance measures. To assess the confidence of peptides, the PSMs were pre-filtered at a PSM-level FDR of 1%. Then based on the “simple principle” (The parsimony principle), identified peptide sequences were assembled into a set of confident proteins. In order to control the rate of false-positive at protein level, a protein FDR at 1%, which was based on Picked protein FDR strategy [17], would also be estimated after protein inference (Protein-level FDR ≤ 0.01). The protein quantification process included the following steps: protein identification, tag impurity correction, data normalization, missing value imputation, protein ratio calculation and statistical analysis.

In this project, we set up 3 groups for comparison: 10% stretch group vs control group, 17% stretch group vs control group, 17% stretch group vs 10% stretch group, respectively. Proteins with Fold change > 1.2 and *P* value < 0.05 were determined as differentially expressed protein. Cluster analysis of differential proteins, as an effective tool for analyzing gene or protein expression data, can discover the function of unknown genes or proteins by clustering genes or proteins. Here, we analyzed the proteins expression patterns in different sample groups by cluster analysis using Euclidean distance and hierarchical algorithm. Gene Ontology (GO) enrichment analysis involved cell components, molecular function and biological processes. In the GO enrichment analysis of differentially expressed proteins (DEPs), hypergeometric tests were used to find out the significantly enriched GO entries by comparing the significantly DEPs with all the identified proteins as the background. Pathway enrichment analysis of detected proteins was performed based on Kyoto Encyclopedia of Genes and Genomes (KEGG) database. The hypergeometric test was used to identify the significantly enriched pathways in the differential proteins against the background of the detected proteins, so as to determine the possible mechanically responsive and signaling pathways involved in the differential proteins.

### Analysis quantitative western blot

The protein concentration was determined by a BCA protein analysis kit (BOSTER Biological Technology co.ltd). Add RIPA lysate (with protease inhibitor and phosphatase inhibitor) to the cells, blow and mix well. After ultrasonic treatment was performed, the cells were cracked on the ice for 30 min followed by centrifugation 10000 g for 10 min. Quantitative analysis was performed using a small amount of protein solution of BCA. The remaining protein solution was added to the protein loading buffer, placed in 100 °C water bathing for 5 min, and then stored the sample at -20 °C.

Add electrophoresis buffer to start loading protein sample. Prepare 0.45NC μm membrane transmembrane with the current value of 150 mA and the transmembrane time of 60 min. Dissolve 5% skim milk powder in TBS-T, place the membrane into blocking buffer, and shake the decolorizing shaker at room temperature for 1.5 h. Membranes were incubated with primary antibodies to glial fibrillary acidic protein (GFAP, 1:1000, BOSTER; United States), Low-density lipoprotein receptor-related protein (LRP6, 1:1000, Abcam, United States), Thrombospondin 1 (THBS1; 1:1000, Abcam, United States) and Tetraspanin (CD81, 1:1400, BOSTER; United States) overnight at 4 °C. After washed, the blots were incubated with secondary antibody to HRP Conjugated AffiniPure Goat Anti-Rabbit IgG (1:5000, BOSTER, United States) at 4 °C for 2 h. ECL chemiluminescence reagent was placed on the membrane, and imaging analysis was performed by the chemiluminescence imaging analyzer (BIO-RAD, United States). The gray value of protein strip was analyzed by ImageJ (National Institutes of Health, United States).

### GEO bioinformatics analysis

The gene-chip data of astrocytes under high intraocular pressure in mice were searched from Gene Expression Omnibus (GEO, https://www.ncbi.nlm.nih.gov/geo/) database. R language limma package (R-4.0.4 for Windows) was used to screen the data for differential gene. The criteria set for differentially expressed genes (DEGs) were Fold change > 1.2 and *P*.value < 0.05. In order to find the biological significance of differential gene expression under different stretch conditions, we used clusterProfler [18] and ggplot2 [19] to perform two functional enrichment annotated clustering analyses: GO classification and KEGG pathway. The screened differential genes and signaling pathways were compared with the results of the rat mass spectrometry assay to screen out the common differential genes and pathways, so as to determine the changes in the expression of mechanics-sensitive proteins and the signaling pathways that may cause certain cellular behaviors under the mechanical stimulations.

### Statistical analysis

Statistical analysis was performed using Statistical Package for the Social Sciences (SPSS, IBM Inc., United States). The normality of all variables was examined using Shapiro–Wilk tests. Significant differences of proliferation and apoptosis were identified between groups using Student’s t test; significant differences of protein expression were identified between groups using one-way ANOVA analysis with Bonferroni correction test. *P* value of less than 0.05 was considered statistical significance.

## Results

### Cell identification

**Fig. 1:**
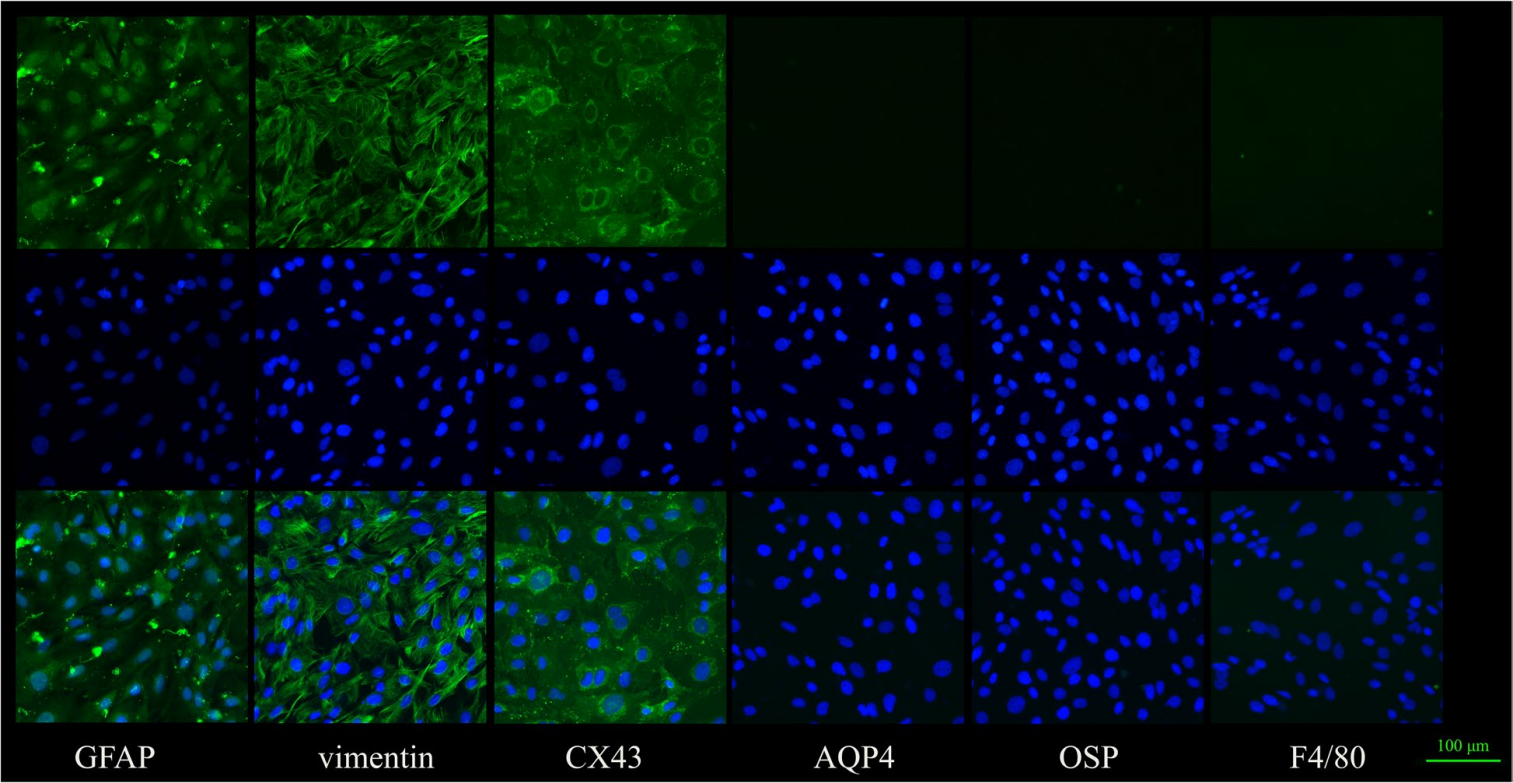
Cell identification results: The results of cell identification showed these cells were astrocytes through positive staining for GFAP, vimentin and Connexin43(CX43). Negative staining for Aquaporin 4 (AQP4), OSP and F4/80 was also shown. Scale bar: 100 μm

GFAP is specifically expressed in the cytoplasm of astrocytes and can be used as an astrocyte-specific marker [20]. In glial cells, vimentin is co-expressed with GFAP. Connexin43(CX43) [21] also expresses in astrocytes and can be a specific marker for identification of astrocytes. Aquaporin 4 (AQP4) [22] is absent from astrocytes in the rodent optic nerve head. F4/80 and OSP [23] label microglia and oligodendrocytes, respectively. These three antibodies react negatively with astrocytes, and can be used as negative markers for ONH astrocytes identification. We chose different areas in the same dish to stain GFAP, vimentin, CX43, AQP4, OSP and F4/80, respectively. The results of cell identification showed that the cells were positive stained by GFAP, vimentin and CX43, and positive cells accounted for more than 95% of the total number of cells respectively. Negative staining for Aquaporin 4 (AQP4), OSP and F4/80 was also shown. These results indicated that the cells were astrocytes (Fig. 1).

### Cytoskeletal arrangement

**Fig. 2:**
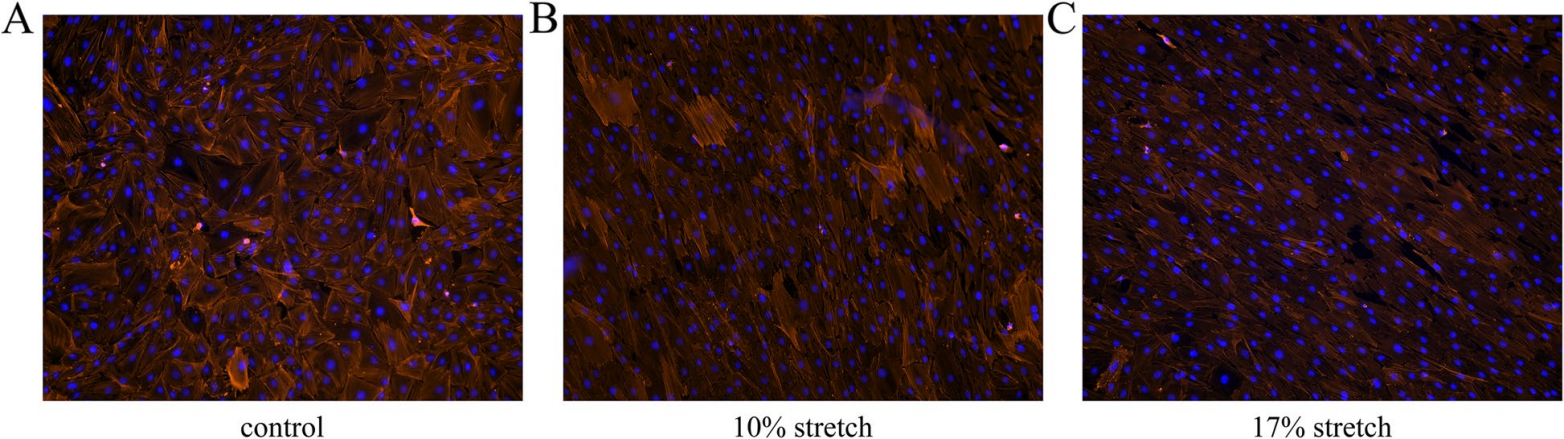
Cytoskeleton changes before and after stretch: (**A**) Cells in the control group were arranged irregularly. (**B**) and (**C**) were cytoskeleton changes after 10% and 17% stretch, in which the astrocyte cytoskeleton was arranged in an orderly manner and the cell shape was fusiform

The cytoskeleton in the control group was arranged irregularly (Fig. 2A). Under 10 and 17% stretch conditions, the astrocytes were arranged in an orderly manner, and the shape of the cells was fusiform (Fig. 2B and C).

### Apoptosis and Proliferation

**Fig. 3:**
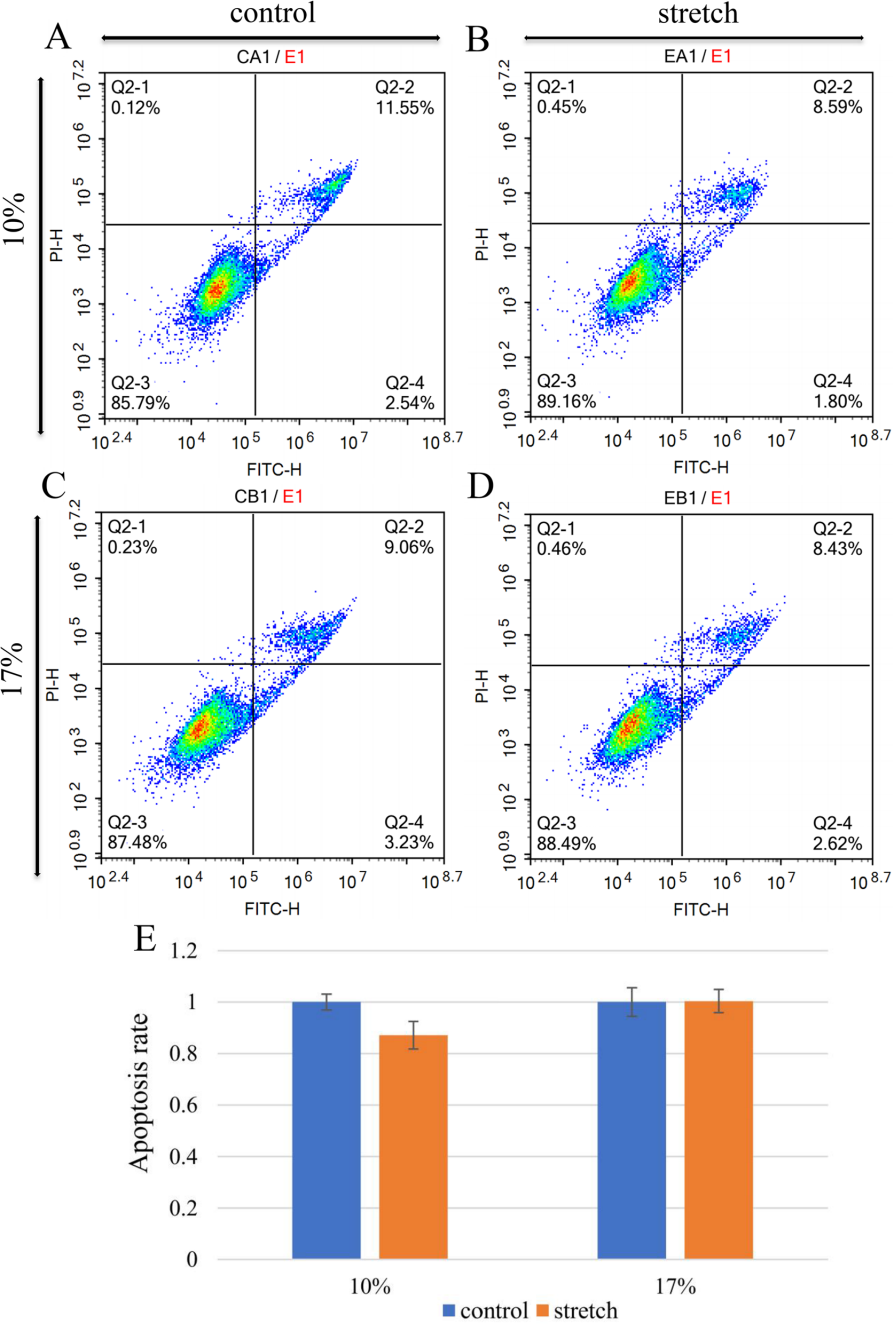
Cell apoptosis before and after stretch: (**A**) 10% control group, (**B**) 10% stretch group, (**C**) 17% control group, (**D**) 17% stretch group. (**E**) The result of apoptosis rate. Error bars indicate standard error of the mean (SEM); there was no statistically significant difference between control and stretch group in both groups

**Fig. 4:**
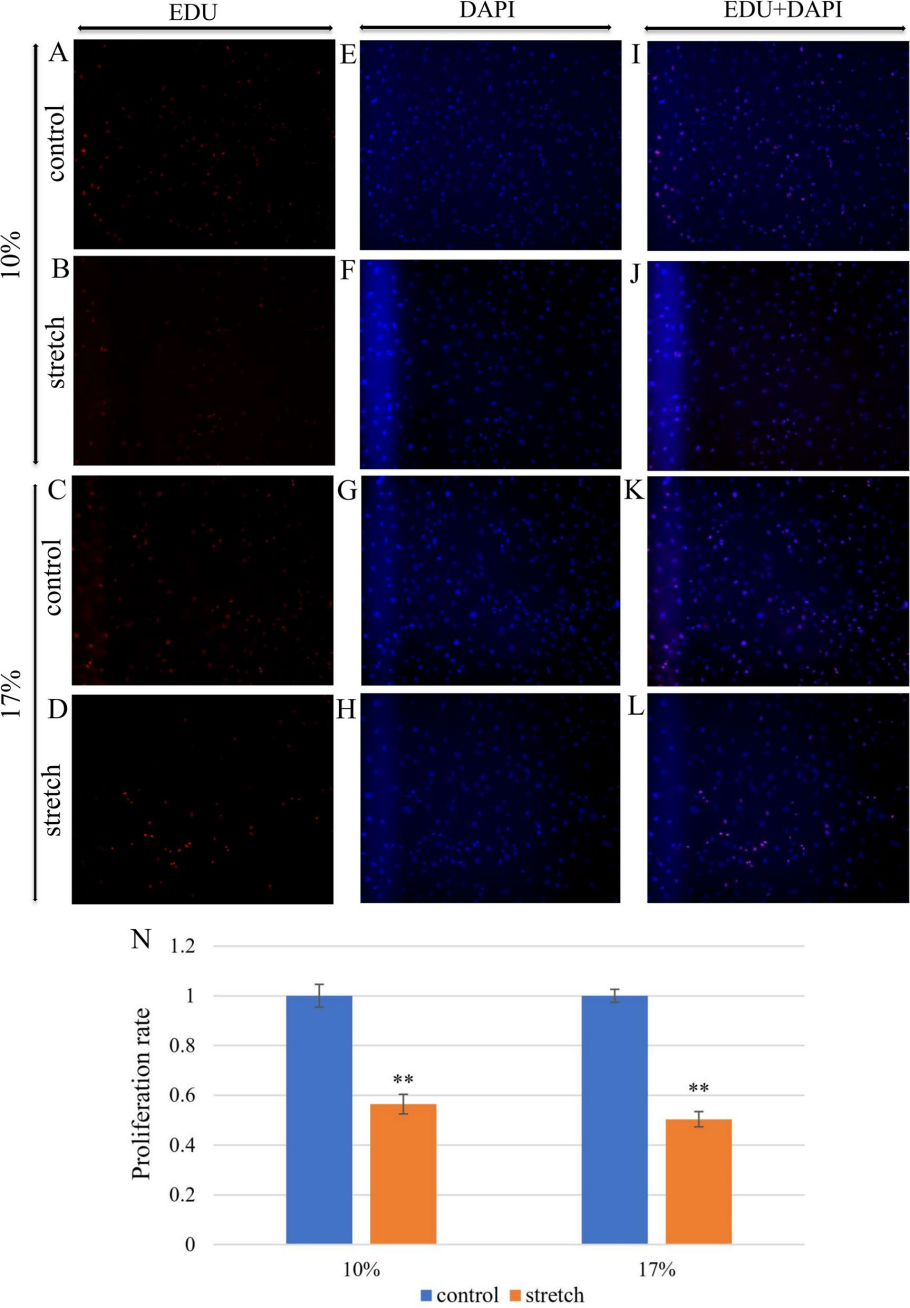
Astrocytes proliferation of different groups: (**A**, **B**, **C**, **D**) EDU labeled astrocytes in 10 and 17% groups. (**E**, **F**, **G**, **H**) DAPI labeled astrocytes in 10 and 17% groups. (**I**, **J**, **K**, **L**) Merged images showed double-labeled astrocytes in all the four groups. (N) Cell proliferation rate decreased in both the 10 and 17% stretch groups. Error bars indicate standard error of the mean (SEM); **P < 0.01 and indicates statistical significance

Flow cytometry results (Fig. 3) showed astrocytes apoptosis rate decreased to a certain extent in 10% stretch group and remained unchanged in 17% stretch group. However, there was no statistical difference in 10 and 17% stretch groups compared with control group. The results of cell proliferation (Fig. 4) decreased in both the 10 and 17% stretch groups.

### Protein identification and protein iTRAQ quantification

In the iTRAQ quantification project, there were 3 groups of samples, which were the control group, the 10% stretch group and the 17% stretch group. We did 3 technique repeats for each group, using Fold Change > 1.2 and *P* value < 0.05 as the screening condition for differential proteins. We considered the protein differentially expressed if more than one of the three results met the conditions. The advantage of this is that we can screen as many differential proteins as we need. Totally 2,903,386 spectrums were generated, 48,973 peptides and 7999 proteins were identified with 1% FDR. 141 DEPs were found in 10% stretch group and 140 DEPs in 17% stretch group compared to control group (Supplementary 1). In order to further select the proteins with significant differences, Fold Change > 1.5 and Fold Change < 0.7 were used as the criteria for up-regulation protein and down-regulation protein, respectively. 13 DEPs and 20 DEPs with significant changes in 10 and 17% stretch groups were selected respectively. Table 1 showed the proteins with the most significant changes in expression after iTRAQ quantification.

**Table 1:** DEPs of interest confirmed by iTRAQ quantitative experiment under two stretch conditions

Protein (gene)	Protein ID	Subcellular localization	Stretch condition	Fold Change*
NACHT and WD repeat domain-containing protein 2-like (LOC103694210)	A0A0G2JW13	ECM	10%24 h 17%24 h	2.23 0.18
Caspase recruitment domain family, member 10 (CARD10)	A0A0G2JTY7	nucleus	10%24 h 17%24 h	0.40 0.39
N-alpha-acetyltransferase 60 (NAA60)	Q3MHC1	cytoskeleton	10%24 h 17%24 h	0.43 0.41
Nuclear receptor subfamily 2 group C member 1 (NR2C1)	Q8VIJ4	nucleus	10%24 h 17%24 h	0.58 0.39

^*^The average value of “Fold Change” obtained from three technical repetitions is shown

### GO and KEGG enrichment analysis of differential proteins

**Fig. 5:**
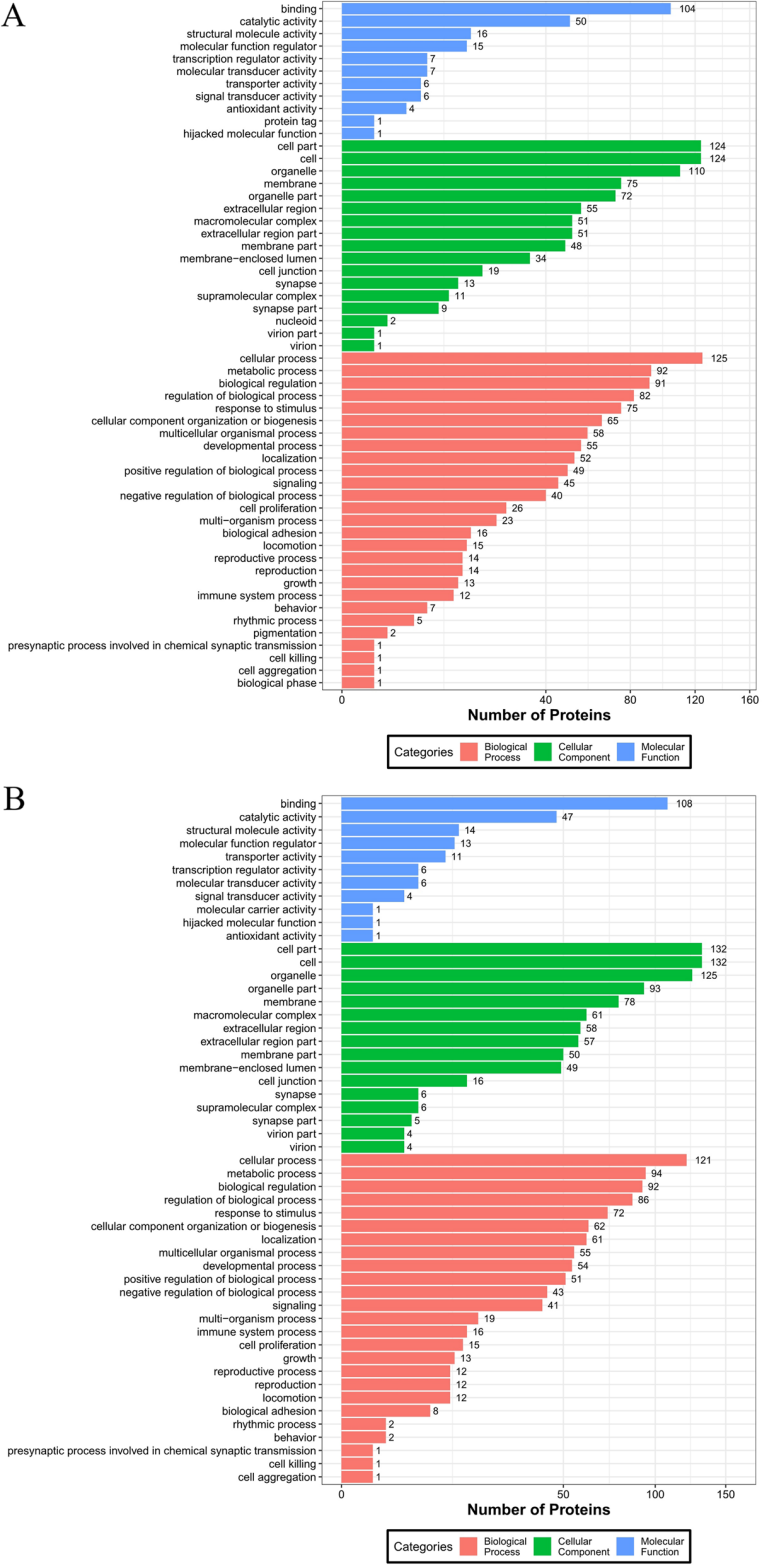
GO of differentially expressed proteins in 10% stretch group (**A**) and 17% stretch group (**B**)

Through GO enrichment analysis, significant DEPs were screened for annotation (*P* < 0.05), as shown in Fig. 5. The 10% stretch group was enriched in 418 protein functions, including 56 molecular functions, 44 cellular components and 318 biological processes. The 17% stretch group was enriched in 373 protein functions, including 52 molecular functions, 67 cellular components and 254 biological processes. Table 2 showed the DEPs were presented under both stretch conditions. These proteins were involved in cell proliferation, signaling transmission, cell apoptosis, cell cycle, cell remodeling and stress response.

**Table 2:** Proteins of interest confirmed by GO analysis under two stretch conditions

Protein (gene)	Protein ID	Subcellular localization	Stretch condition	Fold Change
Low-density lipoprotein receptor-related protein (LRP6)	A0A0G2K0H3	plasmalemm	10%24 h 17%24 h	0.83 0.93
Thrombospondin 1 (THBS1)	Q71SA3	ECM	10%24 h 17%24 h	0.84 0.88
Tetraspanin (CD81)	Q6P9V1	plasmalemm	10%24 h 17%24 h	0.72 0.67

^*^The average value of “Fold Change” obtained from three technical repetitions is shown

Through pathway enrichment analysis, significant DEPs were screened for analysis (*P* < 0.05). The 10% stretch group was enriched in 26 signaling pathways, while the 17% stretch group was enriched in 21 signaling pathways. The results showed that the DEPs were significantly enriched in PI3K-Akt signaling pathway, MAPK signaling pathway, Jak-STAT signaling pathway, ECM receptor interactions, and TGF-β signaling (Table 3).

**Table 3:** Signaling pathways of interest confirmed by KEGG analysis under two stretch conditions

Pathway ID	Signaling pathways	Proteins with pathway annotation (7502)	Related differential protein gene (Fold Change)
ko04151	PI3K-Akt signaling pathway	186 (2.48%)	THBS1 (0.88)
ko04010	MAPK signaling pathway	173 (2.31%)	TGF-β3 (0.81)
ko04310	Wnt signaling pathway	98 (1.31%)	LRP6 (0.83) ANTXR2 (1.85)
ko04630	Jak-STAT signaling pathway	56 (0.75%)	/
ko04512	ECM-receptor interaction	52 (0.69%)	THBS1 (0.88)
ko04064	NF-kappa B signaling pathway	49 (0.65%)	CARD10 (0.4)
ko04350	TGF-beta signaling pathway	44 (0.59%)	TGF-β2 (0.89) TGF-β3 (0.81) THBS1 (0.88)

^*^The average value of “Fold Change” obtained from three technical repetitions is shown

**Fig. 6:**
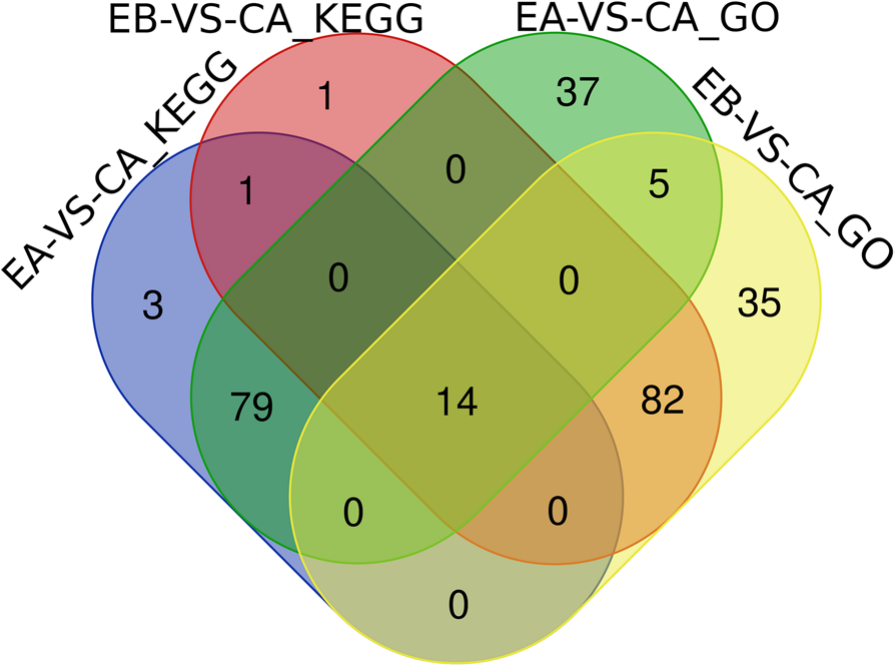
Differential proteins from GO and KEGG enrichment analysis, where CA is control group; EA is 10% stretch group; EB is 17% stretch group

We compared the proteins enriched by GO and KEGG (Fig. 6). A total of 14 proteins were found in both 10 and 17% stretch group of the GO and KEGG enriched protein results. One protein of KEGG results was found in both 10 and 17% stretch groups. And 5 proteins of GO enriched results were expressed in two stretch groups (Table 4). There were 79 proteins found in the GO and KEGG enrichment results obtained from the 10% stretch group, and 82 proteins were found from the GO and KEGG enrichment results obtained in the 17% stretch group.

**Table 4:** Differential proteins from GO and KEGG enrichment analysis

Group	Total	Protein_ID	Gene_ID
EA-VS-CA_GO; EA-VS-CA_KEGG; EB-VS-CA_GO; EB-VS-CA_KEGG	14	tr|A0A0G2JTY7|A0A0G2JTY7_RAT	CARD10
		tr|D4A213|D4A213_RAT	SCARA5
		tr|D3ZD83|D3ZD83_RAT	MFSD10
		sp|P02793|FRIL1_RAT	FTL1
		sp|O70536|SOAT1_RAT	SOAT1
		tr|B0K031|B0K031_RAT	RPL7
		tr|B2RZD4|B2RZD4_RAT	RPL34
		sp|Q62632|FSTL1_RAT	FSTL1
		tr|Q66HI5|Q66HI5_RAT	FTH1
		tr|Q71SA3|Q71SA3_RAT	THBS1
		sp|Q07258|TGFB3_RAT	TGF-β3
		tr|Q6P9V1|Q6P9V1_RAT	CD81
		tr|F1LPM3|F1LPM3_RAT	SORBS2
		tr|A0A0G2K0H3|A0A0G2K0H3_RAT	LRP6
EA-VS-CA_KEGG; EB-VS-CA_KEGG	1	tr|Q923Z2|Q923Z2_RAT	TPM1
EA-VS-CA_GO; EB-VS-CA_GO	5	tr|M0R7B4|M0R7B4_RAT	HIST1H1D
		sp|Q3MHC1|NAA60_RAT	NAA60
		sp|Q8VIJ4|NR2C1_RAT	NR2C1
		sp|P15865|H14_RAT	HIST1H1E
		tr|D3ZY47|D3ZY47_RAT	RGD1559896

### Verification of mass spectrometry experimental results by western blot

**Fig. 7:**
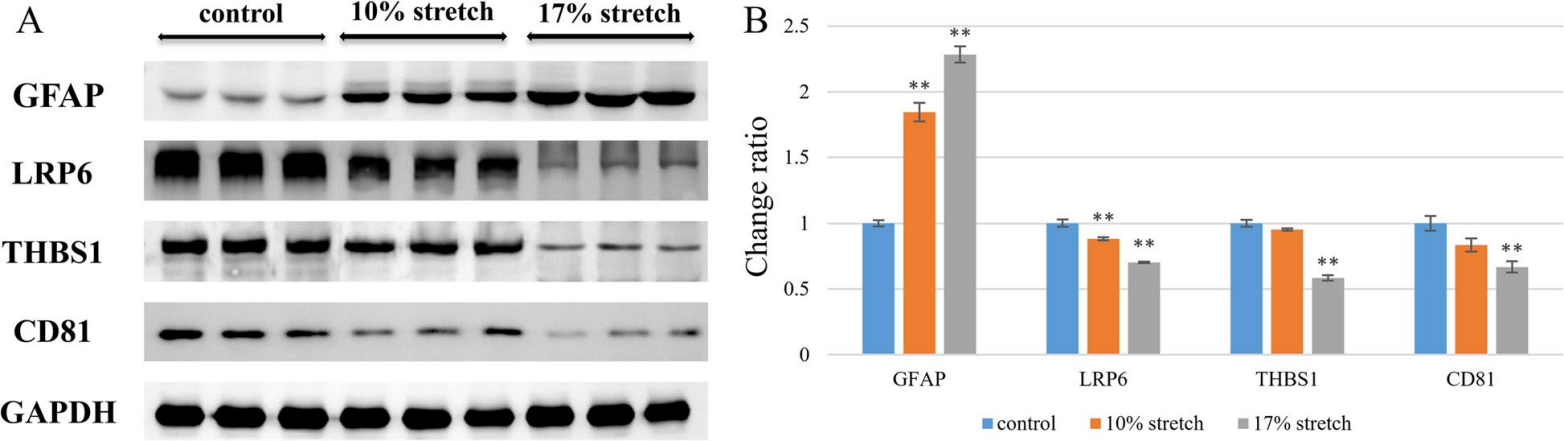
Western blot analysis of specific protein expression (*n* = 3). (**A**) Colored bands of GFAP, LRP6, THBS1 and CD81; (**B**) Expression levels of LRP6, THBS1 and CD81 gradually decreased with the increase of the stretch amplitude, while the expression level of GFAP gradually increased with the increase of the stretch amplitude. Error bars indicate standard error of the mean (SEM); *P < 0.05 and **P < 0.01 indicate statistical significance

Western blot was performed on LRP6, THBS1 and CD81 respectively to verify the protein screening results. We did 3 technique repeats for each group, and normalized the results. The results showed that the expression levels of four proteins of interest gradually decreased with the increase of stretch amplitude. These results were consistent with the results of iTRAQ quantitative experiments. Meanwhile, western blot was used to detect the GFAP expression level. Astrocytes became activated when they were damaged by injury or disease, a notable sign of which was the increase of GFAP expression. The results showed that, compared with the control group, the expression level of GFAP gradually increased with the increase of stretch amplitude (Fig. 7).

### Verification of mass spectrometry experimental results by GEO

Through differential analysis, a total of 465 differentially expressed genes (DEGs) were screened out in GSE40009, of which 180 genes were up-regulated and 285 genes were down-regulated. Compared with mass spectrometry, 2 genes were both differentially expressed, including ANTXR2 and CARD10. Through gene function enrichment analysis, it was found that these genes were mainly concentrated in the biological functions of extracellular matrix remodeling, cell proliferation, cell migration and cell adhesion. As for signaling pathway enrichment, we found that these genes were mainly enriched in regulation of actin cytoskeleton and neurotrophin signaling pathway.

## Discussion and conclusion

Based on the observation of cell phenotypes, we found that the cytoskeleton, apoptosis and proliferation of astrocytes were altered under different amplitudes of stretch, and the effect of mechanical stimulate might lead to the activation of astrocytes. Rearrangement of the actin cytoskeleton in response to stimulation by mechanical stress has been considered to be mechanical responses of the astrocytes [4, 24]. The result of astrocytes apoptosis rate indicated a reduction under 10% stretch condition and stayed unchanged under 17% stretch condition. But there was no statistically significant difference between the control group and two stretch groups. In previous studies, TUNEL labeling indicated occasional astrocyte apoptosis in optic nerve heads of 6-week IOP elevation mice model [25]. This may suggest that the observation of apoptosis requires a longer stretch time. Results of cDNA microarrays [26] and immunohistochemical labeling [27] showed astrocytes proliferation under elevated IOP. The significant astrocytes proliferation has been quantified by Ki67 labeling in rat glaucoma models [9] and in mice 1 week after IOP elevation [25]. After 6 weeks with IOP elevation, the astrocyte nuclear number was the same as that of controls. Our result showed a decrease in the astrocyte proliferation rate, which may be related to the in vitro culture of astrocytes, or the astrocyte proliferation underwent an upward and downward process, which will be further verified in future studies.

Astrocyte activation has previously been associated with the early development of glaucoma optic neuropathy [28, 29]. In this study, we used iTRAQ-based proteomic analysis to investigate differential protein expression in rat ONH astrocytes while the amplitude of stretch was 10 and 17% for 24 h, respectively. The stretch amplitudes were based on finite element models from previous research [30–32]. Voorhees [30] built finite element models of LC microstructure of sheep eyes, and found the deformation of sheep eyes between IOP’s of 0 and 10 mmHg is small when compared with the deformation between 10 and 20 mmHg, and as strains in the range of 10 to 20% can trigger astrocytic remodeling and neuron damage. In the microstructure model, the peak (90th percentile) first principal strains exceeded 15%. Sigal [32] predicted a peak strain within the lamina cribrosa approaching 15% when IOP was raised to 50 mmHg. The 10% strain was used to represent a much lower insult. These studies provided the basis for our experiments. These models directly contributed to the various stretch conditions that were chosen for our experiments. More than 6000 total proteins were found under different stretch conditions. The CV value of coefficient of variation showed the high reliability of iTRAQ technology, which supported the application of this technology in this study and experimental design. The results of the western blot experiment were consistent with that of iTRAQ.

We have observed that some proteins may be responsible for the activation or injury of astrocytes, and the proteins of interest identified based on cluster analysis and GO analysis may have a potential role in astrocyte responses.

Based on ITRAQ experimental results, the protein expression of LRP6 downregulated to 0.83 in the 10% stretch group. We also verified this change in western blot experiments. The LRP6 is a member of the LDL receptor family, a kind of cell surface receptor, which can interact with a variety of ligands. It was also involved in the regulation of axon growth, as an important surface receptor in the Wnt/β–catenin pathway, and played an important role in activating the typical Wnt/β-catenin signaling pathway [33, 34]. XU et al. found that microrna-513c inhibited the proliferation of glioblastoma cells by inhibiting LRP6 [35].

ANTXR2 was differentially expressed both in this study and in the GEO database. In the mass spectrometry experiment, the expression of ANTXR2 upregulated in two of the three comparison groups. The ratio of the protein expression level of 10% stretch group to control group was 1.85, while that of 17% stretch group to 10% stretch group was 1.09. There was no significantly change in the 10% stretch group compared with control group. This result indicated that the expression of ANTXR2 might change only when a certain extent of stretch was reached. ANTXR2 is a membrane-bound molecule that can interact with LRP6. LRP6 is an important surface receptor in the Wnt/β–catenin pathway, and thus can affect osteoblastic activity [36]. In String database (https://string-db.org), we found that ANTXR2 and LRP6 had a co-expression relationship (Supplementary 2). These studies demonstrated that AntXR2 and LRP6 might co-act on the Wnt/β–catenin pathway under the pathological effects of glaucoma.

The expression of CARD10 downregulated both in this study and in the GEO database. In the mass spectrometry experiment, the expression of CARD10 reduced to 0.4 and 0.39 under 10 and 17% stretch conditions, respectively. CARD10 is a member of the caspase recruitment domain (CARD) protein family, which is closely associated with the occurrence of tumors, and its role is mainly to promote tumor progression by activating the transcription factor NF-κB [37]. Studies have confirmed that CARD10 expression is associated with glaucoma and ocular hypertension. In previous Genome-wide association studies (GWAS), CARD10 was significantly associated with optic disc parameters [38] and showed enrichment in POAG samples [39, 40]. These findings indicated CARD10 may contribute to POAG pathogenesis. Tezel et al. [12] analyzed proteomic responses of retinal astrocytes in an experimental rat model, and found inflammatory responses of astrocytes in experimental glaucoma included up-regulation of a number of immune mediators/regulators linked to the activation of NF-κB signaling pathway. Another study also indicated NF-κB signaling pathway played a key role in neuroinflammatory and neurodegenerative outcomes of experimental glaucoma [41]. CARD10 exerts its biological functions mainly through the NF-κB signaling pathway. Some studies also indicated that NF-κB signaling pathway was important in regulating numerous genes involved in cell proliferation, survival, and apoptosis [42, 43].

CD81 protein expression downregulated to 0.67 in the 17% stretch group. CD81 is a member of the Tetraspanin protein family and is thought to be associated with the regulation of cellular behavior. CD81 is associated with reactive gliosis and the glial scar [44] and inhibits the proliferation of astrocytes by inducing G(0)/G(1) arrest in vitro [45]. CD81, also known as a target of the antiproliferative antibody, is expressed in astrocytes and involved in cell adhesion. Song et al. [46] found that the expression of CD81 was related to GFAP, the classic marker of astrocyte activation, both of which were upregulated after retinal injury. We infer that CD81 may be related to the activation of astrocytes, but the mechanism is still not clear.

We verified the downregulation of THBS1 in stretch conditions using western blot experiments. Thrombospondin-1 (THBS1) is a matricellular protein that negatively regulates angiogenesis and inflammation in the eye [47]. It has also been proved to be a protein naturally involved in the activation of latent TGF-β. TGF-β2 is associated with glaucomatous neuropathy, primarily via the increased synthesis and secretion of extracellular matrix (ECM) proteins and remodeling of the optic nerve head (ONH) [48, 49]. TGF-β2 is capable of inducing the expression of ECM and basement membrane components in cultured ONH astrocytes via THBS1 [50]. TGF-β is one of the ways to induce glaucoma models that lead to remodeling of the extracellular matrix. We believe that the mechanisms, even though there are not clear yet, of pharmacological induction and mechanical stimulation remain somewhat different, which may have different effects on cells [51]. Different stimulations all lead to changes in THBS1 expression, suggesting that THBS1 may be one of the influential factors in the pathogenetic development of glaucoma. Further investigations will also be conducted in future studies.

The results of pathway enrichment analysis also suggested some possible mechanosensitive pathways, including PI3K-Akt signaling pathway, MAPK signaling pathway, Jak-STAT signaling pathway, ECM receptor interactions, and TGF-β signaling pathways. PI3K-Akt signaling pathway [52–54] and MAPK signaling [55, 56] pathway play an important role in mediating retinal ganglion cell apoptosis in glaucoma. Early Jak-Stat pathway activation in ONH injured by elevated IOP is associated with astrocyte cell proliferation [9].

In summary, we investigated mechanobiological responses to astrocytes in optic nerve head due to biaxial stretch, including the change of morphology, proliferation, apoptosis, proteins of interest and signaling pathways using mass spectrometry experiments as well as bioinformatics. We have identified a number of proteins of potential interest, including LRP6, CARD10, THBS1, CD81. And signaling pathways include Wnt/β–catenin pathway, NF-κB signaling pathway, PI3K-Akt signaling pathway, MAPK signaling pathway, Jak-STAT signaling pathway, ECM-receptor interaction, and TGF-β signaling pathway, which might associate with astrocyte activation. These findings may contribute to a better understanding of the activation of astrocytes and the role astrocyte activation played in glaucomatous optic neuropathy.

Bioinformatics analysis was used in the hope of increasing the credibility of the experimental results. However, we were unable to find eligible rat data in the GEO database. Therefore, we used the mice database for bioinformatics analysis because rats and mice are homologous and have more overlapping similar genes. We will collect more and stronger evidence to support our results in the subsequent studies. Cell proliferation, apoptosis and cytoskeleton changes are not yet directly linked to the differential genes and enriched signaling pathways we obtained, and we will provide more evidence to support this idea in the future.

Due to the experimental conditions and time constraints, there were some limitations in this study. Firstly, this study only investigated the proteomics of astrocytes under two stretch amplitudes for 24 h. Secondly, we simulated the stress environment of astrocytes in the optic nerve head using Flexcell FX-5000 T tension system, which is different from the stress environment of cells in vivo, such as the differences in the extracellular environment and the three-dimensional environment. Finally, some functions of analyzed proteins and pathways still need to be validated in the next study.

In future studies, we will establish a three-dimensional pressure environment to explore the mechanobiological responses of astrocytes in vitro. Furthermore, we will also establish a rat model with chronic high intraocular pressure to explore the influence factors of astrocyte activation under chronic hypertension. Eventually, we hope to identify proteins of interest and potential biomarkers involved in astrocyte activation.

## Supplementary Information

**Additional file 1.**

**Additional file 2.**

**Additional file 3.**

## Data Availability

The datasets used and/or analysed during the current study are available from the corresponding author on reasonable request.

## Abbreviations

IOP: Intraocular pressure
ONH: Optic nerve head
GEO: Gene Expression Omnibus
LRP6: Low-density lipoprotein receptor-related protein
CARD10: Caspase recruitment domain family, member 10
THBS1: Thrombospondin 1
CD81: Tetraspanin
TGF-β: Transforming growth factor-β
GFAP: Glial fibrillary acidic protein
iTRAQ: Isobaric tags for relative and absolute quantification
RT: Room temperature
GO: Gene Ontology
KEGG: Kyoto Encyclopedia of Genes and Genomes
NF-κB: Nuclear factor kappa-B
