# Supplementary material for: Mechanobiological responses of astrocytes in optic nerve head due to biaxial stretch
Source: BMC Ophthalmol. 2022 Sep 16;22:368. doi: 10.1186/s12886-022-02592-8 (PMC9482189; doi:10.1186/s12886-022-02592-8)
Supplement: Supplementary file 2 — Additional file 2. [file 12886_2022_2592_MOESM2_ESM.docx]

Supplementary Material

# Supplementary 2


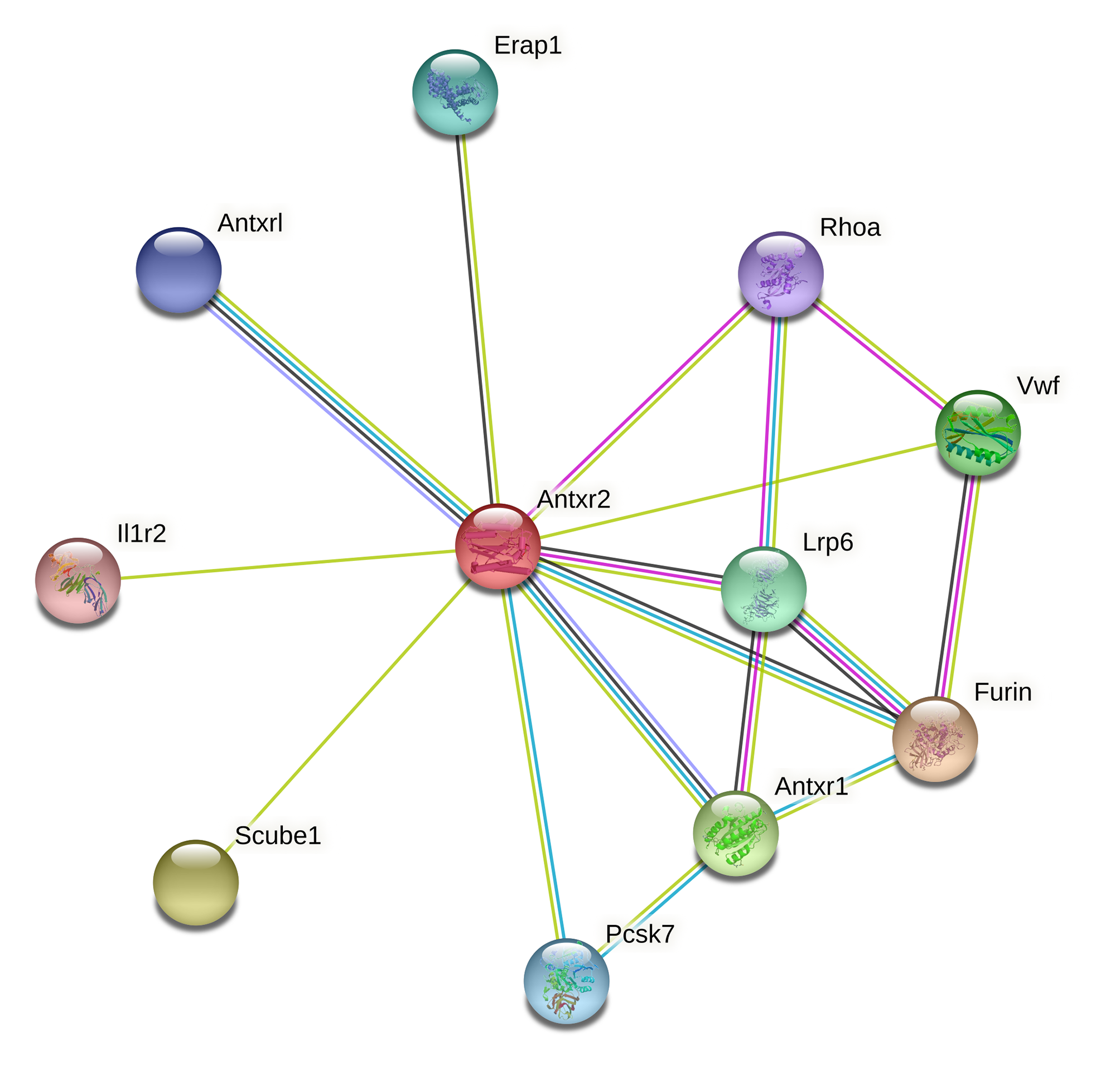


**Supplementary 2** ANTXR2 functional protein association network (https://string-db.org)
