# Supplementary figures and images for: Mechanobiological responses of astrocytes in optic nerve head due to biaxial stretch
Source: BMC Ophthalmol. 2022 Sep 16;22:368. doi: 10.1186/s12886-022-02592-8 (PMC9482189; doi:10.1186/s12886-022-02592-8)

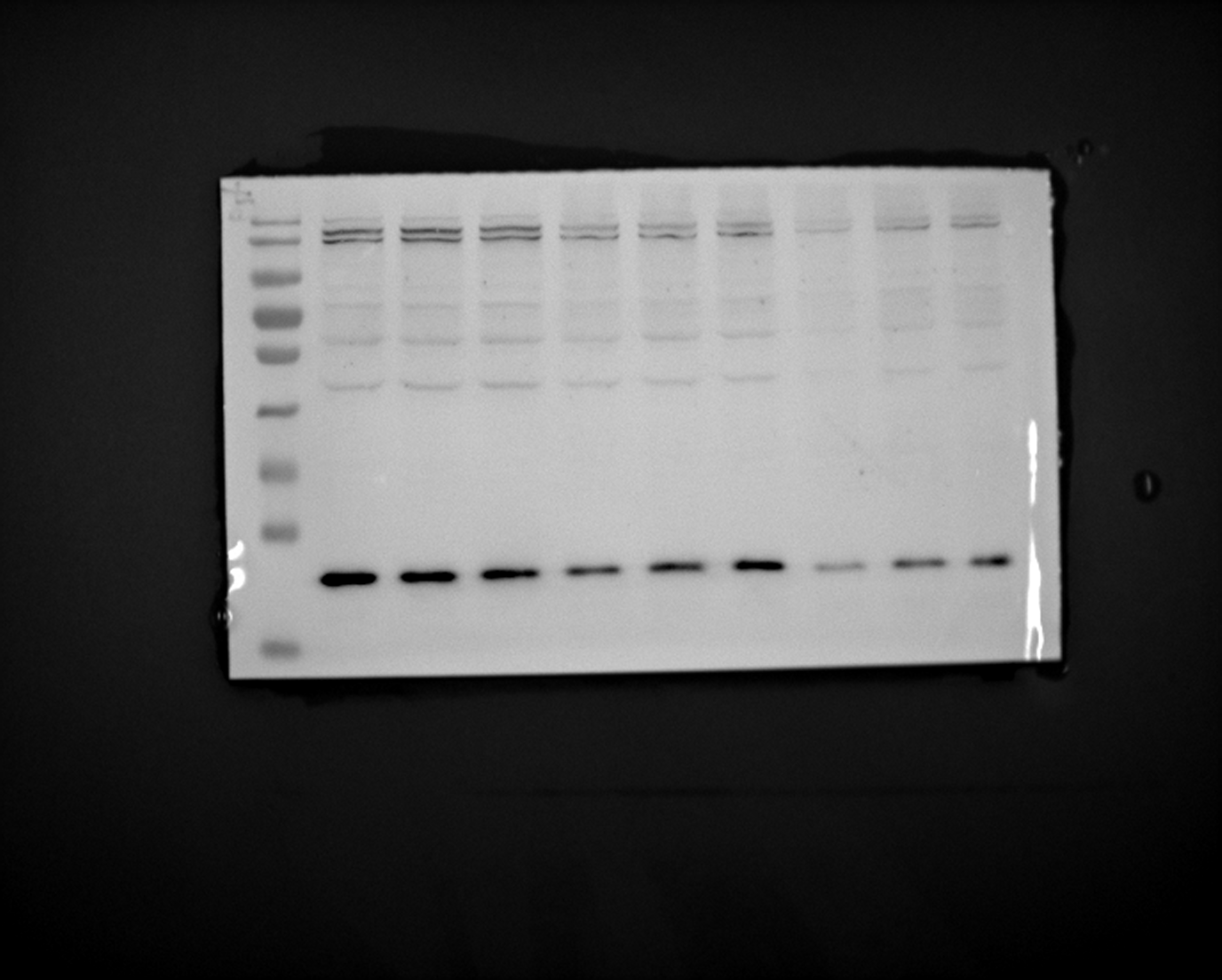

Supplement: Supplementary file 3 — Additional file 3. [file 12886_2022_2592_MOESM3_ESM.zip › Figure7A_WB_CD81.Tif]

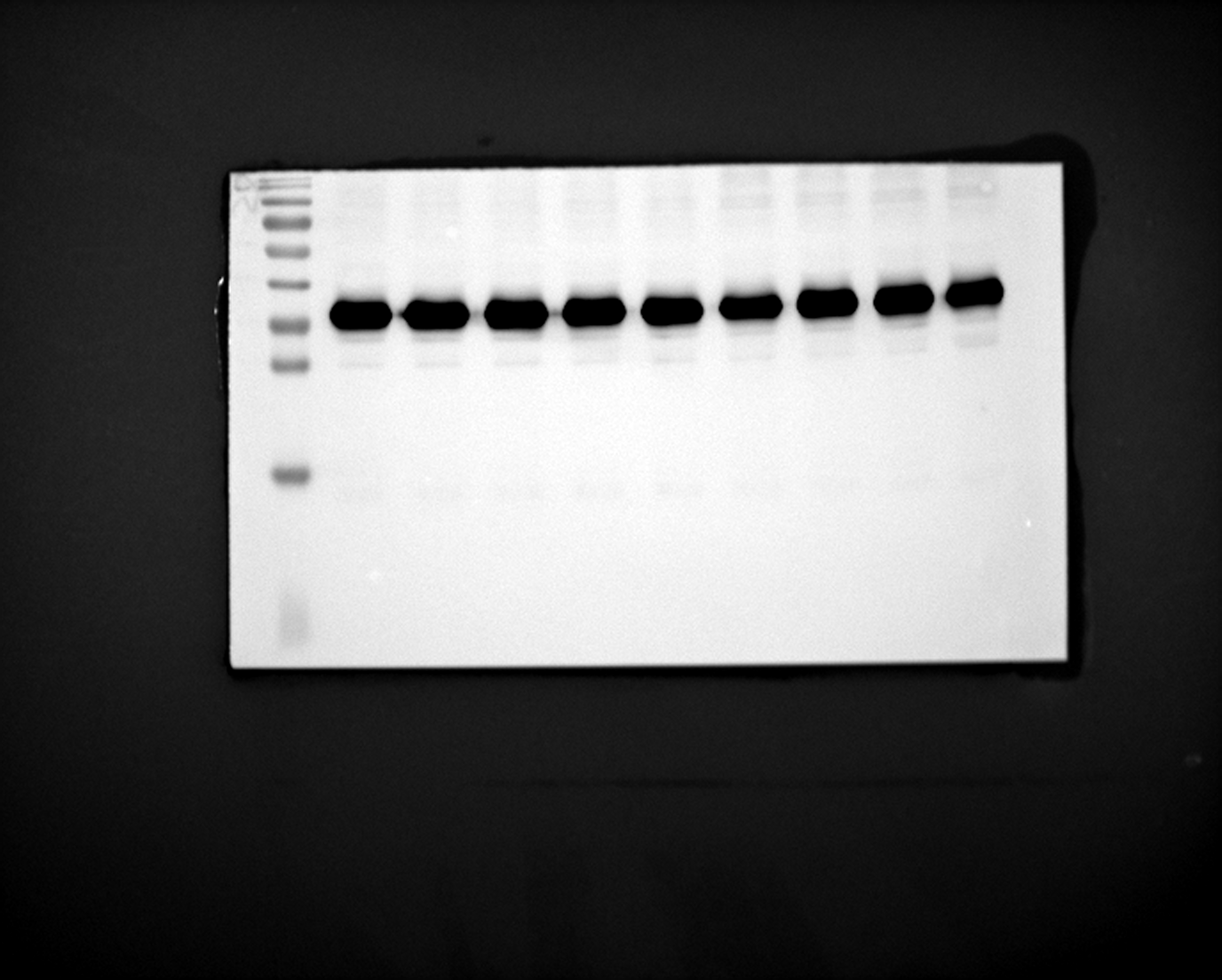

Supplement: Supplementary file 3 — Additional file 3. [file 12886_2022_2592_MOESM3_ESM.zip › Figure7A_WB_Gapdh.Tif]

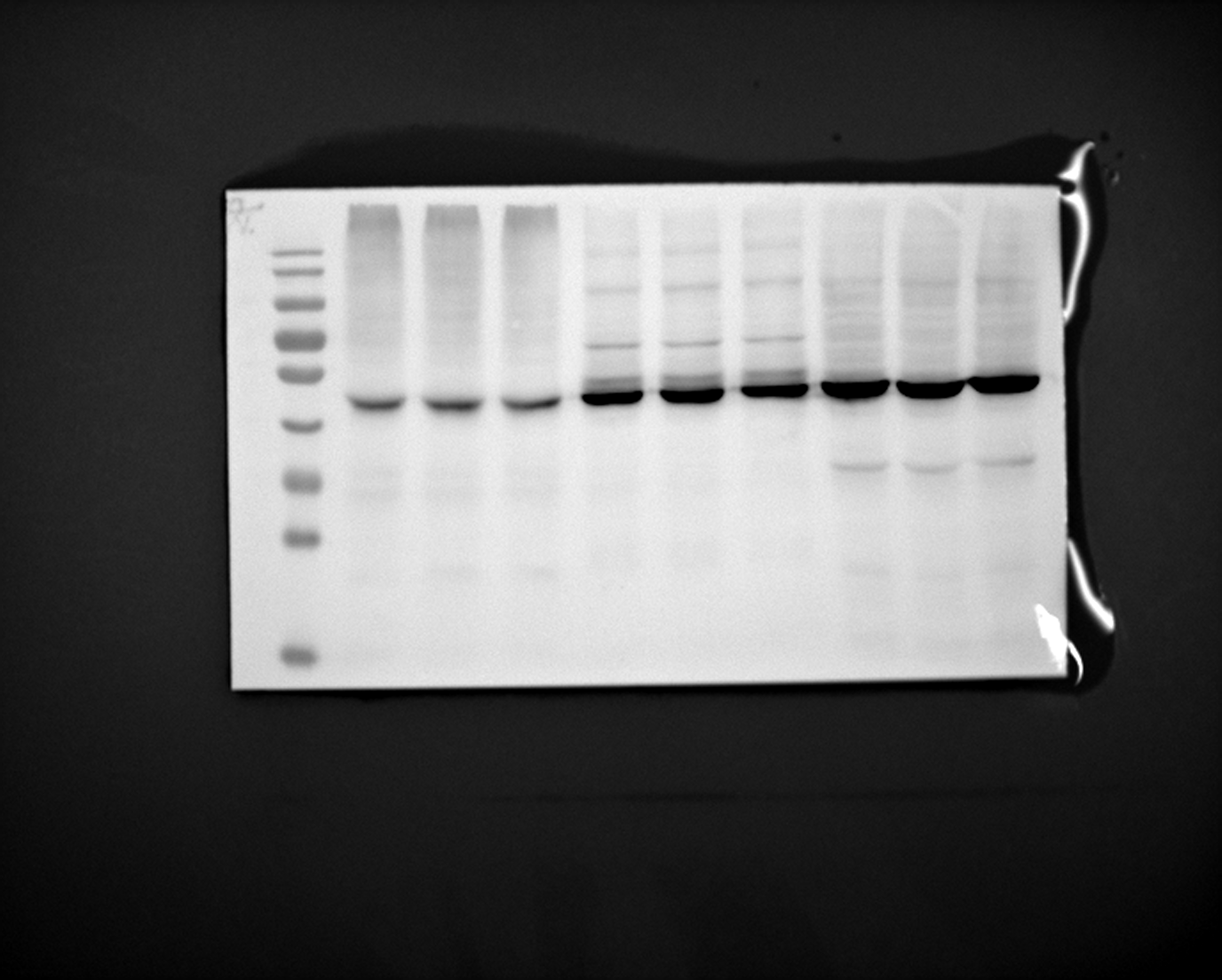

Supplement: Supplementary file 3 — Additional file 3. [file 12886_2022_2592_MOESM3_ESM.zip › Figure7A_WB_GFAP.Tif]

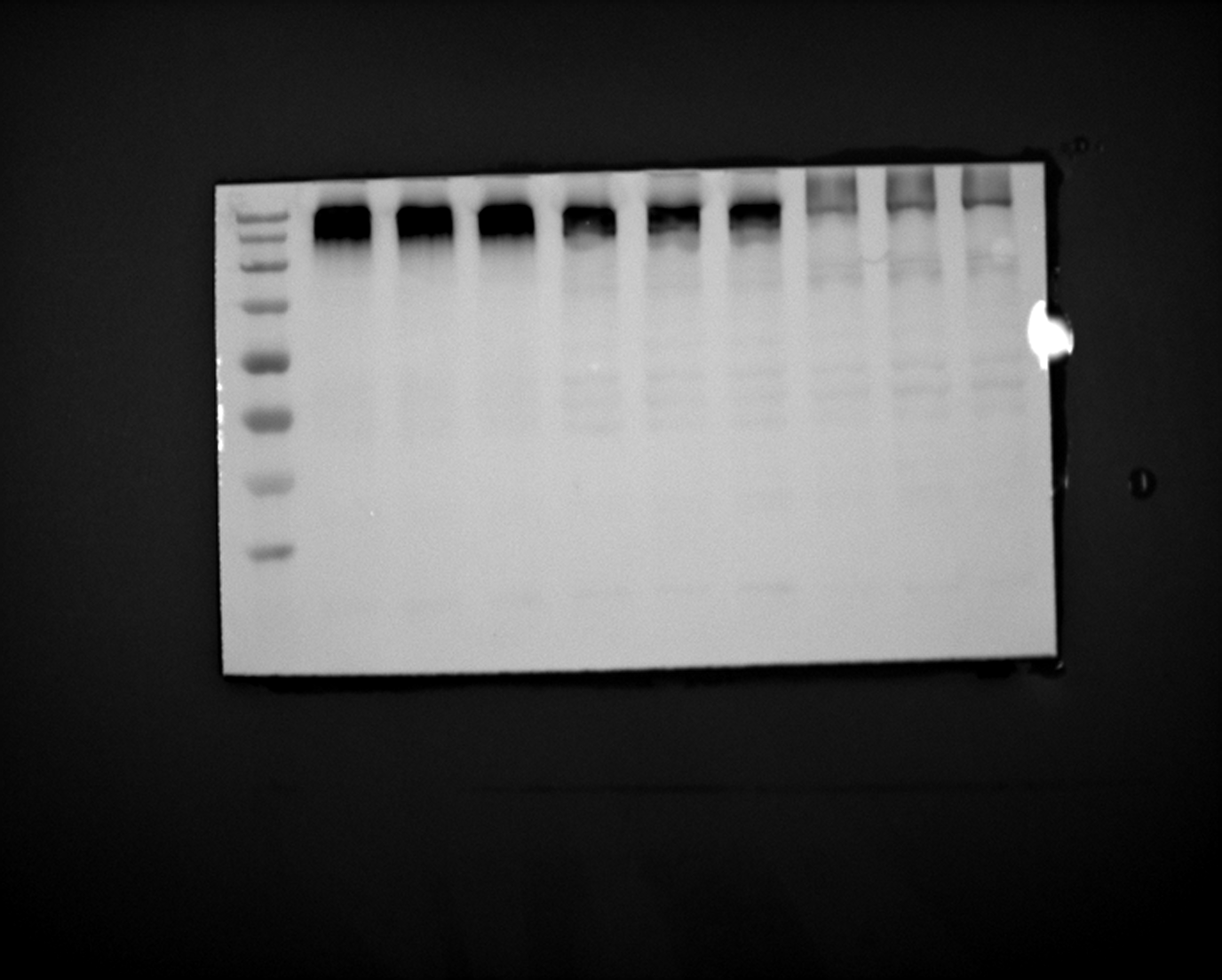

Supplement: Supplementary file 3 — Additional file 3. [file 12886_2022_2592_MOESM3_ESM.zip › Figure7A_WB_LRP6.Tif]

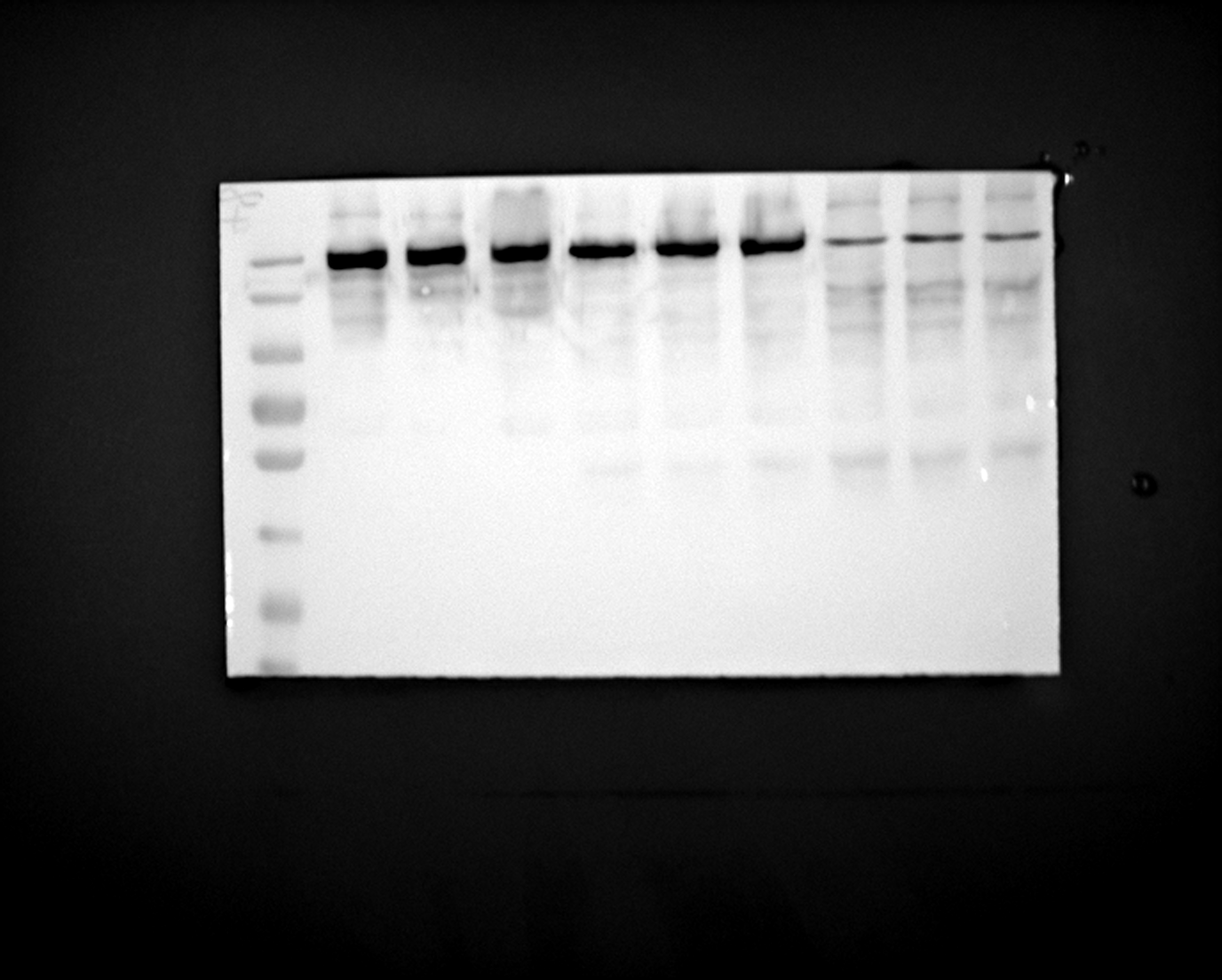

Supplement: Supplementary file 3 — Additional file 3. [file 12886_2022_2592_MOESM3_ESM.zip › Figure7A_WB_THBS1.Tif]
